# Midterm Results of Tension Band Wiring Technique for Acute Rockwood Type III Acromioclavicular Joint Dislocation

**DOI:** 10.7759/cureus.12203

**Published:** 2020-12-21

**Authors:** Fırat Ozan, Sefa Gök, Kürşat Tuğrul Okur, İbrahim Altun, Murat Kahraman, Ali Eray Günay, Kamil Yamak

**Affiliations:** 1 Orthopedics and Traumatology, Kayseri City Hospital, Kayseri, TUR; 2 Orthopedics and Traumatology, İzmir Bozyaka Training and Research Hospital, Izmir, TUR

**Keywords:** acromioclavicular dislocation, tension band wiring, surgical treatment, complication, rockwood

## Abstract

Background

The aim of this study was to evaluate the clinical and radiological results of patients with acute type III Rockwood acromioclavicular joint (ACJ) dislocation treated surgically by employing tension band wiring.

Methods

The study included 24 patients with traumatic type III ACJ dislocations according to the Rockwood classification. The clinical and radiological outcomes of patients were assessed at the final follow-up visit. Implant failure and reduction loss were assessed using radiographs, whereas the Constant-Murley scoring system was used to assess the patients clinically.

Results

The mean follow-up period was 3.5 ± 1.3 years (range: 1-6 years). The mean age of the patients was 41.8 ± 11.7 years (range: 19-64 years) and the mean length of hospital stay was 2.3 days (range: 1-6 days). The fixation material was removed postoperatively at an average of 7.2 ± 9.9 months (range: 3-40 months). At the end of the follow-up period, the mean Constant-Murley score was 72.5 ± 12.8 (range: 50-90). The ACJ reduction was stable in 13 (54.2%) patients. Residual subluxation was detected in 11 (45.8%) patients. Distal clavicular osteolysis was noted in six (25%) patients. Acromioclavicular osteoarthritis was detected in five (20.8%) operated shoulders on follow-up radiographs. During the follow-up, Kirschner-wire migration and breakage occurred in four (16.6%) and seven (29.1%) patients, respectively.

Conclusions

This study showed that surgical treatment with the tension band wiring method provided functionally satisfactory results even if complications developed because of the presence of implants. Independent of age, we can recommend it as the primary treatment method for patients who do not have very high expectations regarding their shoulder function. Additionally, we think that reducing the duration of implant retention will reduce the incidence of complications.

## Introduction

Acromioclavicular joint (ACJ) dislocation is a common injury mostly encountered during sporting activities, with an overall incidence of 3-4 per 100,000 in the general population [1,2]. ACJ dislocation occurs typically in a young male [1-4]. These injuries usually result from a direct blow to the acromion with the arm adducted [2,4-6]. Disruption of the normal anatomy and stability of the ACJ can lead to abnormal shoulder function and chronic pain [4].

Rockwood classification is commonly used for such traumas [4,7]. Type I and II injuries are treated with non-surgical methods [1,2,4,7], whereas type IV, V, and VI injuries are treated surgically [1,2,4,7]. However, the choice of treatment for type III acromioclavicular (AC) injuries is controversial [1,8]. Over 60 surgical techniques have been described, each with its specific advantages and disadvantages; however, the ideal method for acute AC injuries currently remains debatable [1,2,6,7,9].

Tension banding using two AC trans-articular percutaneous Kirschner wires (K-wires) and a cerclage is still used to treat acute grade III ACJ dislocation and remains a simple, fast, and inexpensive procedure [1,3,6]. Many studies have shown that the K-wire technique for ACJ dislocation injuries provides good functional results pertaining to range of motion, strength, and pain [1,3,6,10-12]. Another advantage of tension band wiring treatment includes the anatomical reduction of the ACJ with inspection of the intra-articular disc and direct suture of the ruptured ligaments [1,2]. Conversely, while good results can be obtained with the tension band wiring method, a second surgical procedure for the removal of implants is required [1,12].

The objectives of this study were to evaluate the clinical and radiological results of patients with acute type III Rockwood ACJ dislocation treated surgically by employing tension band wiring. The results were compared with those in the literature to determine the suitability of the tension band wiring method for controversial type III acute ACJ dislocation.

## Materials and methods

This study included a total of 24 patients (18 men and 6 women) with traumatic type III acute ACJ dislocation (12 right shoulders and 12 left shoulders) according to the Rockwood classification [7] treated surgically by tension band wiring stabilization technique. The characteristics of the patients are presented in Table 1.

**Table 1:** Demographic characteristics and clinical outcomes of patients

Patient Characteristics	
Number of patients n	24
Age, years, mean (range)	41.8 (19–64)
Sex, n (%)	
Female	6 (25)
Male	18 (75)
Follow-up, years, mean (range)	3.5 (3–40)
Side of involvement, n (%)	
Right	12 (50)
Left	12 (50)
Mean length of hospitalization, days, mean (range)	2.3 (1–6)
Mechanism of trauma, n (%)	
Traffic accident	8 (33.3)
Simple fall	16 (66.7)
Mean length of hospitalization, days, mean (range)	2.3 (1–6)
Time of removal of implants, months, mean (range)	7.2 (3–40)
Constant–Murley score, mean (range)	72.5 (50–90)

Inclusion criteria were acute trauma (<three weeks after trauma), unilateral, isolated type III ACJ dislocation, and patients between 18 and 70 years of age. Patients with signs of osteoarthritis, chronic ACJ dislocation, history of previous ACJ surgical procedure, other traumas in the same arm, systemic diseases, open fracture-dislocations, and pathological fracture-dislocations were excluded.

Surgical technique

**Figure 1:**
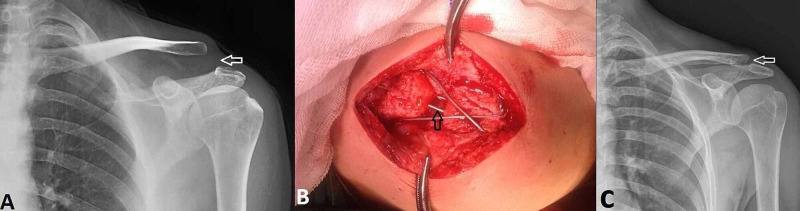
Patient with Rockwood type III acromioclavicular joint dislocation (A) Anteroposterior radiographic images of the joint show a dislocation of the acromioclavicular joint of the left shoulder (white arrow). (B) Intraoperative view of the acromioclavicular joint after reduction and fixation with tension band wiring technique (black arrow). (C) Radiographic image of the patient's left acromioclavicular joint 3 years after surgery (white arrow).

The operative procedure was performed under general anesthesia and beach chair position with the injured limb freely mobile. A 6-cm-long superior longitudinal incision was made perpendicularly to the ACJ. The delto-trapezial fascia and muscle were split longitudinally. The ACJ including the articular surfaces, disk, and ligaments was examined. The joint was cleansed, and the meniscus was left in place if considered intact and removed otherwise. After reducing the ACJ, tension band wiring with two 2.5-mm cross K-wires from the lateral acromion edge into the clavicle and 1.5-mm steel wire in a figure of eight configuration was performed (Figure 1).

The coracoclavicular ligaments were not sutured. To prevent proximal K-wire migration, the lateral pin ends were bent. The correct K-wire and ACJ positions were verified with intraoperative radiographic examination. Finally, the delto-trapezial fascia and muscle, subcutaneous tissue, and skin were sutured.

Postoperative care

A shoulder arm sling was applied for three weeks. Passive shoulder motion by means of pendulum exercises was initiated on the second day postoperatively. Actively assisted shoulder exercises were started three weeks postoperatively till return to normal daily activities. Patients were advised to refrain from elevating the upper limb above the horizontal until six weeks postoperatively. The fixation materials were planned for routine removal at an average of three months postoperatively.

Clinical assessment

The clinical and radiological outcomes of patients were assessed at the final follow-up visit. Complications such as implant failure and loss of reduction were evaluated with radiographs. The patients were assessed clinically according to the Constant-Murley scoring system, including pain, capability of motion, and function (0-55 points, poor; 56-70 points, moderate; 71-85 points, good; 86-100 points, excellent) [13].

## Results

The mean follow-up period was 3.5 ± 1.3 years (range: 1-6 years). The injuries were caused by traffic accidents and falls in 8 (33.3%) and 16 (66.7%) patients, respectively. The mean age of the patients was 41.8 ± 11.7 years (range: 19-64 years) and the mean length of hospital stay was 2.3 days (range: 1-6 days). The fixation material was removed at an average of 7.2 ± 9.9 months postoperatively (range: 3-40 months). At the end of the follow-up period, the mean Constant-Murley score was 72.5 ± 12.8 (range: 50-90).

Complications

**Figure 2:**
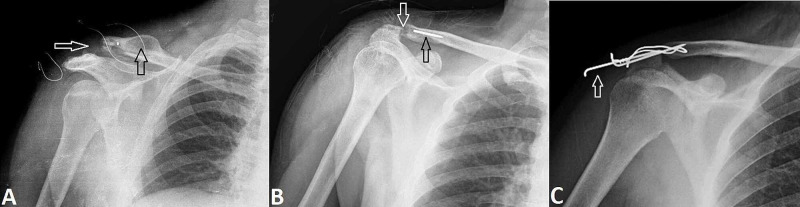
Radiographic images of complications Anteroposterior radiographic images of the shoulder show (A) residual dislocation (white arrow), osteolysis in the distal of the clavicle, and hypertrophic bone formation (black arrow), (B) distal clavicular osteolysis (white arrow) and Kirschner-wire breakage (black arrow), and (C) Kirschner-wire migration (white arrow).

The complications observed during the follow-up of the study were mostly related to the use of implants (Figure 2).

The ACJ reduction was stable in 13 (54.2%) patients, while residual subluxation was detected in 11 (45.8%) patients. Distal clavicular osteolysis was noted in 6 (25%) patients. AC osteoarthritis was detected in five (20.8%) operated shoulders on follow-up radiographs. During the follow-up, K-wire migration and breakage occurred in four (16.6%) and seven (29.1%) patients, respectively.

No infectious or neurovascular complications were detected (Table 2).

**Table 2:** Complications reported during the study

Radiographic findings	n (%)
Residual subluxation	11 (45)
Distal clavicular osteolysis	6 (25)
Acromioclavicular osteoarthritis	5 (20)
Kirschner-wire migration	4 (16)
Wire breakage	7 (29)

## Discussion

In this study, we evaluated the results of the surgical treatment in which we applied AC fixation with tension band wiring in patients with type III acute ACJ dislocation. At the conclusion of the study, good clinical results were obtained. However, various complications arising from implants were detected during the follow-up.

Many studies have both favored and countered the need for surgical treatment that is commonly indicated for type III acute ACJ dislocation in adults [6,8,9]. The appropriate indication of treatment for this grade of lesion is still a matter of discussion in orthopedic surgery [6,8,9]. However, many authors advocate surgical treatment [1,3,4,8,10,11,14].

It is generally accepted that high-grade ACJ dislocation leads to long-term pain and disruption [3-5,10]. Therefore, there is consensus regarding the acute surgical management of Rockwood type IV, V, and VI dislocations that is approved by the validated data obtained from multiple studies [4,5,14].

Problems such as residual pain and weakness have been reported after non-operative treatment of Rockwood grade III ACJ dislocations [3,4,14]. Surgery is recommended to avoid potential shoulder deformity and poor functional outcomes, especially in young active individuals [4,8,14,15].

Surgical treatments offer improved outcomes and functionality [5,14] compared with that of non-surgical modalities or surgical treatments that are performed more than three months after the injury [9]. The management of type III injuries is individualized for patients [3,5,10,14,15].

Gumina et al. have proposed that unreduced, complete ACJ dislocations may impair the normally coupled scapuloclavicular mechanics due to the loss of a stable shoulder fulcrum, resulting in scapular dyskinesis and pain [16].

Although there are no clearly defined protocols, surgical treatment is recommended in young patients with overhead activity, those working in jobs requiring strength, those whose clavicle is locked superiorly and posteriorly to the acromion, and those with cosmetic deformity and shoulder stiffness [4-8,11,12,15]. The non-surgical treatment seems to be the preferred choice for elderly patients [6,7,10,14].

The best surgical option for ACJ reconstruction remains unclear [4,5,9,10,15]. Various surgical methods have been described such as the Weaver-Dunn technique, coracoclavicular suture technique, ACJ ligament repair technique, cerclage slings, screw fixation, plate fixation methods, free graft reconstruction, arthroscopically assisted ligament reconstruction, and various special suturing techniques [2,5,10,15]. However, the learning curve for some techniques is long and complex [6]. Despite the many surgical techniques described, none of these techniques have been shown to be clinically superior to another [4,9,15]. Each surgical technique has its own unique complications [4,9,15].

One of the most frequently performed surgical technique to treat acute grade III ACJ dislocation is temporary transfixation of the ACJ with tension band wiring [3]. Tension band wiring for the ACJ stabilization remains a simple and cost-eﬀective procedure [1,3,6]. An important advantage of the tension band wiring treatment method is the effective repair of delto-trapezial muscles and fascia and AC ligaments [1]. These structures play a very important role in stabilizing the joint [1]. This provides effective control of the horizontal and vertical stability of the clavicula [1].

AC fixation or primary coracoclavicular fixation is generally considered for acute cases (<tree weeks) in which the injured ligamentous structures are believed to have healing potential [4,15]. Biologic coracoclavicular ligament reconstructions are generally recommended in chronic (>three weeks) cases [4,15] and are utilized by some surgeons for all types of reconstructions [4].

Some authors have advocated performing coracoclavicular stabilization with open suturing after ACJ stabilization [1,17,18]. However, Lateur et al. argued against the suturing of coracoclavicular ligaments as they found reducing and stabilizing the joint separation to be sufficient in bringing the ligament ends into contact with each other, thereby ensuring healing [1]. In our study, we operated on our patients during the acute period, within three weeks after the trauma, and we too did not suture the coracoclavicular ligament.

In a follow-up study of 21 years, Lizaur et al. reported that Rockwood type III ACJ dislocations treated with K-wire fixation and suturing of the ACJ ligaments had a satisfactory result in 92% of patients [11].

The study of Leidel et al. demonstrated that surgical treatment with temporary articular transfixation of acute Rockwood type III ACJ dislocations using K-wires enabled good and reliable therapy results with a promising functional outcome in the long term [10]. The mean Constant-Murley score was 88, with no significant differences after 1-2 years, 3-5 years, and 6-10 years [10]. The complication rate was 15% with K-wire migration and recurrent ACJ dislocation in 4% and 11% of cases, respectively [10].

When Lateur et al. compared the results of their tension band wiring study with a multi-center prospective case series treated arthroscopically, they found identical functional results and a lower complication rate [1].

The temporary tension band wiring technique requires a second surgical procedure to remove the wires six to eight weeks after the first procedure [3,6,7,10,11]. The principal complications of tension band wiring method occur early often due to K-wire dislocation or breakage [6]. In our study, we retained the wires for a longer time to avoid the risk of secondary dislocation. We think that this caused a higher rate of complications due to the implants. Although the planned implant removal time was determined as three months, the average implant removal time of the patients in our study was seven months due to various reasons. However, good results were obtained in terms of the functional score.

Biz et al. observed the complications mainly related to K-wire migration (27%), K-wire breakage (10%), and metalwork intolerance (15%), along with ACJ arthritis or osteolysis of the lateral part of the clavicle (13%) [6]. The authors achieved a 87.4% and 90.2% Constant-Murley score in the tension band wiring group and percutaneous trans-acromial K-wire group, respectively [6].

Lateur et al. reported that K-wire migration occurred in four (16%) patients and caused recurrent ACD in two (8%) patients in a follow-up study of 12.5 years [1]. They detected distal clavicular osteolysis in 2 (8%) patients and AC osteoarthritis in eight (47%) patients [1]. Among the latter, only 25% had symptoms [1]. Functionally good or very good results were achieved in 84% of the patients [1].

Clinical results are often not correlated with radiological findings [3]. Detectable residual ACJ dislocation or posttraumatic arthrosis does not appear to be associated with functional treatment outcomes [3,19,20]. In their study, Taft et al. detected 35% posttraumatic ACJ arthrosis radiographically and reported no effect on clinical results [19]. Among the 30 patients evaluated by Rawes and Dias, all had residual ACJ dislocations or subluxations 12.5 years following an ACJ type III injury; nevertheless, 29 patients had good functional outcomes [20]. In the 21-year follow-up study of Lizaur et al., radiologically, there were five patients with redisplacement of the ACJ; three had satisfactory results, whereas two reported unsatisfactory results due to moderate pain [11]. At the end of the study, AC osteoarthritis signs were detected in 28% of the patients [11]. Among the patients with severe osteoarthritis, one had unsatisfactory results because of redisplacement and pain [11]. They reported that coracoclavicular ossification was seen in 10 patients, none of whom had pain [11]. In our study, ACJ residual subluxation was detected in 11 (45.8%) patients, distal clavicular osteolysis was noted in six (25%) patients, and AC osteoarthritis was detected in five (20.8%) operated shoulders on follow-up radiographs. ACJ redislocation was not detected. The infection rate after surgical treatment in ACJ dislocations has been reported to be 15% [2]. No infection developed in any of the patients in our study.

While AC joint stabilization with tension band wiring method provides low morbidity, easy application, and safe fixation, complications such as fixation loss, fracture, and loosening can be seen in the internal fixation material used [3,14]. Therefore, it may require a second surgical intervention [3,10,14,15]. In our study, during the follow-up, K-wire migration and breakage occurred in four (16.6%) and seven (29.1%) patients, respectively.

Our study had some limitations. First, the study did not have a control group and its results were not compared with those of the other surgical methods. Second, the patients were followed-up for a limited duration.

## Conclusions

This study demonstrated satisfactory functional outcomes with the surgical treatment by using tension band wiring in acute Rockwood type III ACJ dislocation. Functionally satisfactory results can be obtained even if complications develop due to implants. Independent of age, we can recommend it as the primary treatment method for patients who do not have very high expectations regarding their shoulder function. The complications observed during the follow-up period were related to the use of implants, such as implant migration, implant failure, osteolysis, distal clavicular arthrosis, and residual subluxation. Additionally, we think that retaining the implants for a shorter duration will reduce the incidence of complications.
